# Clinical Trial Protocol for LPS-Boost: Intensified [^177^Lu]Lu-PSMA-617 Treatment for Patients with Metastatic Castration-Resistant Prostate Cancer with Low PSMA–Expressing Disease

**DOI:** 10.2967/jnumed.126.272472

**Published:** 2026-10

**Authors:** Amir Iravani, Roman Gulati, Robert Miyaoka, Angela Castellanos Reiger, Delphine L. Chen, Meena Kumar, Mark Erickson, Katie Nega, Catherine Meyer, Stephanie Lira, Youngho Seo, Robert R. Flavell, Collin C. Pritchard, Michael C. Haffner, Rosa Nadal Rios, Rajitha Sunkara, Hiba M. Khan, Todd Yezefski, Ruben Raychaudhuri, Andrew C. Hsieh, Petros Grivas, Heather H. Cheng, Evan Y. Yu, Michael T. Schweizer, Peter S. Nelson, Johannes Czernin, Thomas A. Hope, Jeremie Calais

**Affiliations:** 1 Department of Radiology, University of Washington, Seattle, Washington;; 2 Clinical Research Division, Fred Hutchinson Cancer Center, Seattle, Washington;; 3 Public Health Sciences Division, Fred Hutchinson Cancer Center, Seattle, Washington;; 4 Department of Nuclear Medicine and Theranostics, University of California Los Angeles, Los Angeles, California;; 5 Department of Radiology and Biomedical Imaging, University of California San Francisco, San Francisco, California;; 6 Department of Laboratory Medicine and Pathology, University of Washington, Seattle, Washington;; 7 Department of Medicine, Division of Hematology Oncology, University of Washington, Seattle, Washington; and; 8 Division of Human Biology, Fred Hutchinson Cancer Center, Seattle, Washington

**Keywords:** ^177^Lu-PSMA-617, radiopharmaceutical therapy, metastatic castration-resistant prostate cancer, PSMA expression, PSMA PET

## Abstract

[^177^Lu]Lu-PSMA-617 is an approved therapy for metastatic castration-resistant prostate cancer (mCRPC); however, response rates vary significantly, particularly among patients with low prostate-specific membrane antigen (PSMA) expression on PSMA PET. This multicenter, open-label phase 2 trial evaluates the efficacy and safety of an intensified Lu-PSMA treatment schedule in patients with mCRPC and a whole-body tumor SUV_mean_ of less than 10 on [^68^Ga]Ga-PSMA-11 PET, a subgroup associated with poorer outcomes with the conventional treatment schedule. **Methods:** Eligible participants (*n* = 45) with mCRPC, prior exposure to androgen receptor pathway inhibitor therapy, and progressive disease (defined by the Prostate Cancer Working Group 3 criteria) must have PSMA-positive lesions without PSMA-negative sites and a whole-body tumor SUV_mean_ of less than 10 on [^68^Ga]Ga-PSMA-11 PET within 3 mo of enrollment. Participants will receive up to 6 cycles of [^177^Lu]Lu-PSMA-617 (7.4 GBq per cycle; cumulative dose, 44 GBq) administered at an intensified cadence (days 1 and 8, days 50 and 57), followed by 2 standard cycles every 6 wk. The primary endpoint is prostate-specific antigen (PSA) progression-free survival, with safety as a key secondary endpoint, assessed in accordance with the Common Terminology Criteria for Adverse Events version 5.0. A continuous safety monitoring rule allows early termination if grade 3 or higher adverse hematologic events (excluding lymphopenia) exceed predefined thresholds established by historical data. Additional secondary endpoints include PSA response, radiographic progression-free survival, overall survival, and patient-reported outcomes (Functional Assessment of Cancer Therapy-Prostate, Brief Pain Inventory—Short Form). Exploratory analyses include radiation dosimetry and plasma biomarker assessments. Participants achieving a marked response (≥90% PSA decline to <1 ng/mL or no PSMA-avid disease above liver uptake on 24-h posttreatment SPECT/CT) after the intensified schedule may pause treatment and resume in the event of PSA or clinical progression. **Conclusion:** By modulating treatment intensity using baseline PSMA expression, this trial aims to improve outcomes in patients predicted to have lower response rates under the standard schedule while maintaining acceptable toxicity.

## Visual Abstract

**Figure:**
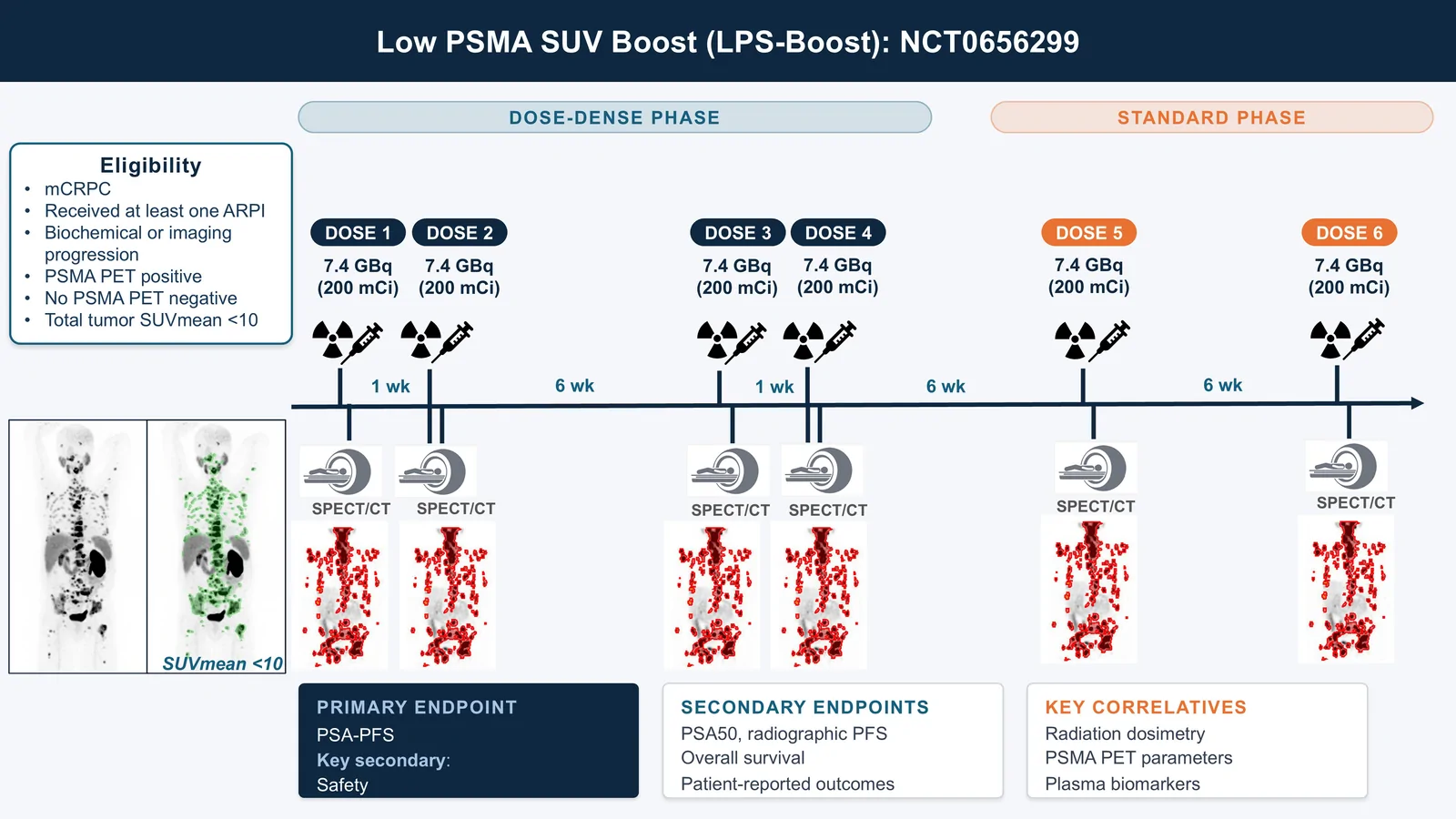

In the past decade, an increasing number of approved therapies with various mechanisms of action have become available for metastatic castration-resistant prostate cancer (mCRPC), but the associated improvements in overall survival (OS) are modest and not generally cumulative (1). It is of interest to develop therapeutic strategies based on emerging biomarkers to increase the benefit from currently available treatments while limiting treatment-related toxicities.

Therapy with [^177^Lu]Lu-PSMA-617, a prostate-specific membrane antigen (PSMA)–targeted radiopharmaceutical therapy, resulted in an improvement in OS in the VISION trial after taxane chemotherapy and improved progression-free survival (PFS) in the PSMAfore trial before the use of taxane chemotherapy and has become an established treatment in patients with mCRPC (2,3). PSMA PET serves as a gatekeeper for [^177^Lu]Lu-PSMA-617 therapy eligibility, and correlative analyses from multiple trials, including TheraP and VISION, have shown that higher PSMA expression on PET imaging is associated with better outcomes (4–6). In the TheraP trial, participants whose whole-body tumor SUV_mean_ on PSMA-targeted PET was in the highest quartile demonstrated a reduction of at least 50% in prostate-specific antigen level (PSA50) compared with those in the lower 3 quartiles of SUV_mean_ (91% vs. 52%) (4). Longer radiographic PFS (rPFS) and prostate-specific antigen (PSA)–PFS were also noted in those with an SUV_mean_ of 10 or greater on PSMA-targeted PET compared with the lowest 3 quartiles (rPFS, 12.7 mo vs. 6.0 mo; PSA-PFS, 8.9 mo vs. 4.5 mo) (4). Consistent results were noted in the VISION trial, where those in the highest quartile of PSMA PET uptake (SUV_mean_, ≥10.2 for rPFS and ≥9.9 for OS) had significantly longer rPFS and OS, compared with the lower 3 quartiles (14.1 vs. 5.8 and 21.4 mo vs. 14.5 mo, respectively) (5). Although these comparisons highlight a greater magnitude of benefit among patients within the highest quartile of PSMA expression, they also underscore the urgent need for strategies to improve outcomes in those within the lower 3 quartiles of PSMA expression, who represent most of this patient population (4,5).

Current treatment protocols for [^177^Lu]Lu-PSMA-617 use fixed administered activity and fixed intervals, disregarding substantial variability in tumor biology, including target expression. Clinical studies indicate that early treatment cycles deliver a higher absorbed radiation dose to tumors (7,8). The observed diminishing returns in subsequent cycles suggest that early cycles carry greater therapeutic weight, supporting the rationale for front-loading treatment when target expression is highest. We hypothesized that reducing the interval between [^177^Lu]Lu-PSMA-617 administrations from 6 wk to 1 wk, early in the course of treatment, allows front-loading of the treatment when PSMA expression is at its highest, while preserving the safety profile.

## MATERIALS AND METHODS

### Study Design

This is an open-label, multicenter, single-arm, phase 2 trial evaluating an intensified [^177^Lu]Lu-PSMA-617 treatment schedule in participants with mCRPC and a whole-body tumor SUV_mean_ of less than 10 on [^68^Ga]Ga-PSMA-11 PET. The study is being conducted across 3 academic centers: the Fred Hutchinson Cancer Center/University of Washington, University of California Los Angeles, and University of California San Francisco, and the protocol has been approved by the institutional review boards of all participating institutions. All participants must provide written informed consent. The trial opened to accrual on February 7, 2025, and is expected to complete recruitment 18 mo after opening. This trial is registered at ClinicalTrials.gov (NCT06526299)

### Study Population

Participants are eligible for study inclusion if they have mCRPC, an Eastern Cooperative Oncology Group performance status of 0–2, progressive disease (in accordance with the Prostate Cancer Working Group 3 criteria), and received treatment with at least 1 androgen receptor pathway inhibitor (9) (Supplemental Table 1, available at http://jnm.snmjournals.org). The initial version of the protocol required that the participants had prior treatment with taxane-based chemotherapy, but after the Food and Drug Administration’s expansion of the labeling to chemotherapy-naïve patients, this requirement was removed in October 2025. Participants must also have a positive [^68^Ga]Ga-PSMA-11 PET/CT scan with no PSMA-negative lesions within 3 mo of enrollment. Additionally, patients must have a whole-body tumor SUV_mean_ of less than 10 on a [^68^Ga]Ga-PSMA-11 PET/CT scan, analyzed using a previously published semiquantitative approach with MIM software (10). All imaging analysis will be supervised by the nuclear medicine site investigator, who has been trained on the software (supplemental materials).

### Endpoints and Assessment

The primary endpoint of the study is PSA-PFS under an intensified schedule of [^177^Lu]Lu-PSMA-617 administration. PSA-PFS was chosen as a pragmatic endpoint for a single-arm phase 2 trial. The key secondary endpoint is the safety of the intensified treatment schedule in accordance with the Common Terminology Criteria for Adverse Events version 5.0 (CTCAE 5.0). Other secondary endpoints include PSA50, rPFS, OS, objective response rate, and patient-reported outcomes by Functional Assessment of Cancer Therapy–Prostate and Brief Pain Inventory—Short Form. Exploratory endpoints of the study include correlative imaging biomarkers, posttreatment radiation dosimetry, and peripheral blood samples analysis for circulating tumor DNA and immune cell profiling.

Safety laboratory assessments, PSA values, and adverse event assessments (graded per CTCAE 5.0) will be performed every 3 wk and on each treatment administration day during the treatment phase and every 12 wk (±2 wk) after completion of treatment (Supplemental Tables 2 and 3). Imaging, including CT and bone scan, will be scheduled every 12 wk (±2 wk) until disease progression. Patient-reported outcomes measures will be collected at baseline, during each cycle, and at follow-up. The trial participants will undergo quantitative SPECT/CT 24 ± 6 h after each [^177^Lu]Lu-PSMA-617 administration. An additional quantitative SPECT/CT will also be performed at 168 ± 6 h after the first and third [^177^Lu]Lu-PSMA-617 administrations but before the second and fourth [^177^Lu]Lu-PSMA-617 administrations (Fig. 1). Blood samples for translational studies are collected at baseline during each cycle, 6 wk after the final cycle, or at the time of PSA, radiographic, or clinical progression, whichever occurs first. Patients are followed for up to 2 y after treatment for long-term safety and survival.

**FIGURE 1.**
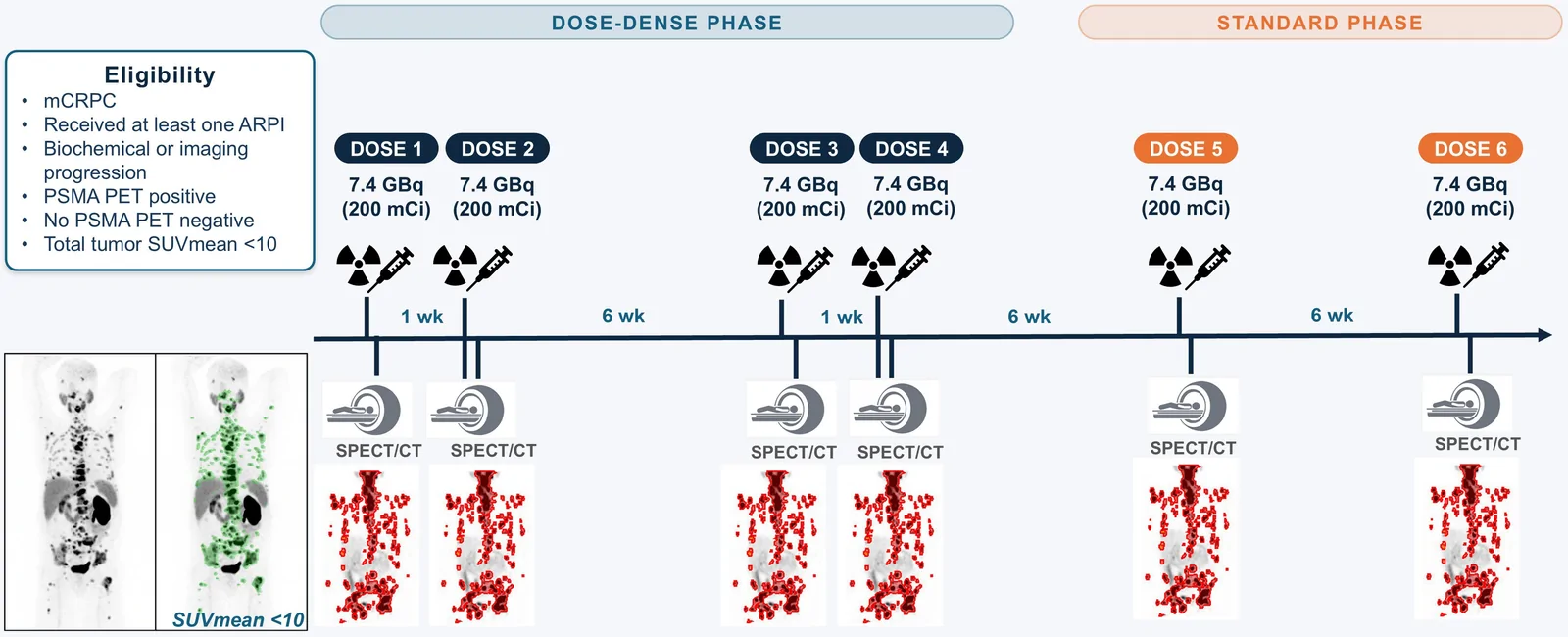
Trial schema. ARPI = androgen receptor pathway inhibitor.

### Treatment Schedule

Patients receive up to 6 cycles of [^177^Lu]Lu-PSMA-617 (7.4 GBq per cycle; cumulative, 44 GBq). The treatment is administered at an intensified cadence (days 1 and 8, days 50 and 57), followed by 2 additional cycles, with a standard 6-weekly cadence (Table 1; Fig. 1).

**TABLE 1.** [^177^Lu]Lu-PSMA-617 Treatment Schedule

Cycle	Day	Dose (GBq)
1	1	7.4
2	8	7.4
3	50 ± 3*	7.4
4	57 ± 3*	7.4
5	99 ±7	7.4
6	141 ±7	7.4
Total		44

* Cycles 3 and 4 must be exactly 7 d apart and can move together within the 3-d window.

### Safety and Monitoring Plan

Dose modifications and delays follow predefined hematologic and biochemical thresholds adopted from the prescribing information. If the criteria for delaying or modifying the administered activity are met during the intensified period of treatment (first 4 cycles), the subsequent cycles must be administered at 6-week intervals once the adverse event has resolved to baseline or grade 1 CTCAE 5.0.

The trial has an integrated early-stopping rule. The study can be terminated early if the proportion of patients experiencing grade 3 or higher adverse hematologic events (except lymphopenia) significantly exceeds 25%, on the basis of historical data from the VISION and TheraP trials (Supplemental Table 4).

A data and safety monitoring board has been established for this trial in accordance with the approved charter, which outlines the composition, responsibilities, and procedures for safety data review and trial oversight as approved by the institutional review boards, which allow early termination via the early-stopping rule.

Treatment may be suspended for marked response, defined as a reduction of at least 90% in PSA level from baseline to a PSA value of less than 1 ng/mL on or after day 71 ± 5 or near-complete loss of PSMA avidity on 24 h [^177^Lu]Lu-PSMA-617 SPECT/CT after treatment, defined as no disease site with uptake above liver background after completion of cycle 4. The treatment can be restarted in the event of biochemical or clinical progression at the investigator’s discretion. The treatment can be discontinued for unacceptable toxicity or radiographic progression or at the investigator’s discretion.

### Statistical Plan

The primary endpoint, PSA-PFS, will be analyzed using a 1-sample log-rank test comparing the observed hazard ratio to the historical control (TheraP trial SUV_mean_, <10 subgroup). In the TheraP trial, the median PSA-PFS for patients with an SUV_mean_ of less than 10 was 4.5 mo. Assuming that this outcome followed an exponential survival distribution, this median corresponds to a hazard ratio of 0.154. If the intensified administration of [^177^Lu]Lu-PSMA-617 achieves a hazard ratio of 0.65 relative to the regimen in that trial—corresponding to a hazard ratio of 0.100 or median PSA-PFS of 6.9 mo—then the anticipated enrollment of 45 patients will provide 80% power to reject the null hypothesis that hazard ratio equals 1 in favor of the alternative hypothesis that hazard ratio is less than 1, using a 1-sample log-rank test with a 1-sided α of 5% (11).

For the key secondary endpoint of safety, we hypothesize that the proportion of patients with CTCAE 5.0 grade 3 or higher adverse hematologic events in the intensified schedule will not exceed the historical data from the VISION or TheraP trials. If the study does not terminate early, then the maximum proportion can be 16 of 45 (36%), with a 90% CI of 25%–48%. Thus, in this worst-case scenario, this study design will provide useful precision about the magnitude of grade 3 or higher adverse hematologic events that is consistent with this regimen (supplemental materials).

## DISCUSSION

This trial aims to address a critical unmet need in mCRPC management: improving outcomes for patients with low PSMA expression, defined as those in the lower 3 quartiles of PSMA expression on [^68^Ga]Ga-PSMA-11 PET, which represents most candidates for [^177^Lu]Lu-PSMA-617 therapy, who exhibit relatively poor responses under the standard treatment schedule. Prior analyses from the TheraP and VISION trials confirm that whole-body tumor SUV_mean_ on PSMA-targeted PET strongly predicts treatment efficacy, with an SUV_mean_ of less than 10 associated with lower PSA50 response rates and shorter PFS, rPFS, and OS compared with patients with an SUV_mean_ of 10 or greater.

Our approach of intensifying the treatment in early cycles leverages multiple principles. First, radiation dosimetry studies have consistently demonstrated a cyclic decline in tumor absorbed dose across successive treatment cycles, particularly after the first 2 cycles (7,8). In a phase 1 study of [^177^Lu]Lu-rhPSMA-10.1, absorbed doses decreased by approximately 37% in cycle 2 and 56% in cycle 3 compared with cycle 1. Pharmacokinetic modeling supports early-cycle dose intensification, administering 50%–100% higher activity during cycles 1 and 2, to increase cumulative tumor dose by 15%–34% without elevating normal organ exposure (12). Various methods of dose intensification have been used in clinical trials. In the TheraP trial, higher administered activity was used at the first cycle (8.5 GBq), with a reduction of 0.5 GB at each subsequent cycle (6). STAMPEDE2, a phase 3 randomized trial, uses a similar approach to this trial by reducing the intervals of cycles for patients with metastatic hormone-sensitive prostate cancer starting androgen deprivation therapy (NCT06320067) (13). Reducing the interval between early cycles, especially the first 2, applies the principle of front-loading and may concentrate tumor radiation delivery while PSMA expression remains high. Furthermore, front-loading could confer protective benefits for normal organs, particularly in patients with a high disease burden (14,15). As tumor volume and target expression decline during treatment, greater radiopharmaceutical availability for organs at risk may increase off-target exposure, reinforcing the rationale for early-cycle intensification.

Second, preclinical studies have demonstrated that PSMA expression can be upregulated in response to DNA-damaging therapies, including ionizing radiation, in a dose-dependent manner within 1–2 wk after treatment (16). This observation provides a rationale for reducing the interval between treatment cycles to approximately 1 wk, thereby leveraging the transient window of increased PSMA expression for enhanced radiopharmaceutical uptake. To evaluate the clinical impact of this phenomenon, integrated posttreatment dosimetry was incorporated into the design. Recognizing the logistic challenges of multiple-time-point dosimetry, which can be resource-intensive for both staff and patients, the protocol simplified imaging to 2 time points. The delayed imaging time point was scheduled at 168 h after administration to coincide with the subsequent treatment cycle, limiting the number of additional visits while enabling accurate dose estimation.

Third, reducing the interval between treatment cycles may exploit several radiobiologic principles to enhance tumor control by limiting the time available for sublethal DNA damage repair, cellular repopulation, and the emergence of resistance mechanisms (17). Cellular senescence is a key mechanism of action for β-particle radiopharmaceutical therapy, but its effects are time-dependent and complex (18). In early stages, successfully treated tumors exhibit sustained γ-H2AX accumulation relative to [^177^Lu]Lu retention during the critical 5–7-d window, primarily driven by delayed induction of senescence rather than apoptosis (19). During this phase, senescent cancer cells activate interferon signaling and upregulate major histocompatibility complex class I expression, promoting robust antitumor immunity mediated by dendritic cells and CD8-positive T cells, which transiently suppresses tumor growth. However, prolonged senescence leads to the accumulation of senescence-associated β-galactosidase and secretion of proinflammatory cytokines, such as interleukin-6), fostering tumor progression, invasion, metastasis, angiogenesis, and immune evasion through recruitment of myeloid-derived suppressor cells and T-cell exhaustion (18,20). These observations underscore the dual nature of senescence in cancer biology and highlight the potential of rapid-sequence radiopharmaceutical administration to harness its beneficial effects while mitigating adverse consequences, a concept warranting further investigation.

The safety of the intensified regimen is a critical endpoint in this trial. Prior evidence from a phase 1–2 dose-escalation study demonstrated that fractionated dosing of [^177^Lu]Lu-PSMA-617 is both safe and effective (21). In that study, 44 men—without preselection on the basis of PSMA PET findings—received administered activities ranging from 7.4 to 22.2 GBq, fractionated on days 1 and 15. No dose-limiting toxicities were observed at the highest tested dose. PSA response was achieved in 61% of patients overall and 71% at the 22 GBq level, with median OS of 16 mo, after only 2 cycles of treatment. Toxicity rates were acceptable, with xerostomia (61%, all grades), fatigue (29%), anemia (25%), and thrombocytopenia (25%) being the most common adverse events. Building on these findings, the current trial incorporates a continuous safety-monitoring approach. The trial is designed to allow early termination if the percentage of patients experiencing grade 3 or higher adverse hematologic events (excluding lymphopenia) significantly exceeds a predefined threshold derived from historical data in major clinical trials.

This multicenter investigator-initiated trial represents one of the few collaborative efforts among academic institutions in the United States to advance radiopharmaceutical therapy. The implementation of such trials revealed several challenges and opportunities. A major barrier has been the lack of dedicated funding mechanisms for radiopharmaceutical research, which historically has limited the scope and pace of clinical investigations. The Society of Nuclear Medicine and Molecular Imaging addressed this gap through the “Mars Shot” initiative, providing critical support for early-phase studies; however, broader engagement with federal agencies, philanthropic organizations, and industry partners remains essential for sustainable progress. This trial underscores the potential of academic–industry partnerships to address clinically relevant questions and accelerate innovation in the field. Furthermore, the absence of a centralized coordinating body for U.S. academic sites highlights an unmet need for structured collaboration. Drawing inspiration from the successful Australasian Radiopharmaceutical Trials Network, the Society of Nuclear Medicine and Molecular Imaging’s Therapy Clinical Trials Network has initiated standardization efforts, including harmonized SPECT camera calibration, which were implemented in this trial. These steps represent foundational progress toward building a robust infrastructure for multicenter radiopharmaceutical trials.

One of the key limitations of this study is its single-arm design, with reliance on historical controls for the statistical evaluation of PSA-PFS. Furthermore, after the expansion of the labeling for [^177^Lu]Lu-PSMA-617 to include chemotherapy-naïve patients in March 2025, the protocol was amended to permit enrollment in the prechemotherapy setting to sustain recruitment. This modification may introduce heterogeneity in the study population, particularly with respect to baseline disease characteristics and expected PSA-PFS, thereby limiting the interpretability and generalizability of the trial outcomes. However, despite these limitations, the observed findings may provide sufficient justification to support the design and conduct of a subsequent randomized controlled trial.

## CONCLUSION

If successful, this trial could establish a new paradigm for biomarker-modulated treatment intensification in theranostics, paving the way for adaptive dosing strategies informed by PSMA expression. Biomarker-guided front-loading of [^177^Lu]Lu-PSMA-617 therapy represents a rational approach to improve outcomes among patients with mCRPC while maintaining an acceptable safety profile. The findings will provide critical insights for future trials that integrate imaging, dosimetry, and molecular biomarkers to personalize radiopharmaceutical therapy and advance precision oncology in prostate cancer.

## DISCLOSURE

Funding for this trial was provided by the Society of Nuclear Medicine and Molecular Imaging Mars Shot Grant (MSRF2023-1). Imaging and therapeutic investigational product and supplemental funding was provided by Novartis (eGC1:A202240). Statistical planning was funded in part by the National Cancer Institute (awards P50CA097186 and R50CA221836). The funding agencies did not have a role in the design or implementation of the trial. No other potential conflict of interest relevant to this article was reported.

## Notes

KEY POINTS **QUESTION:** Is intensified [^177^Lu]Lu-PSMA-617 effective and safe in patients with low PSMA–expressing mCRPC? **PERTINENT FINDINGS:** We describe the protocol for the LPS-Boost trial, an investigator-initiated, single-arm, multicenter phase 2 trial of an intensified regimen of [^177^Lu]Lu-PSMA-617 in participants with mCRPC and a whole-body tumor SUV_mean_ of less than 10 on PSMA-targeted PET. In this trial, intensified cycles are administered by reducing the intervals between cycles 1 and 2 and 3 and 4 to 1 week. **IMPLICATIONS FOR PATIENT CARE:** This trial could establish a paradigm for biomarker-modulated treatment intensification in PSMA theranostics.
